# Present-day land subsidence risk in the metropolitan cities of Italy

**DOI:** 10.1038/s41598-025-18941-8

**Published:** 2025-10-07

**Authors:** Francesca Cigna, Roberta Paranunzio, Roberta Bonì, Pietro Teatini

**Affiliations:** 1 Institute of Atmospheric Sciences and Climate, National Research Council, Via del Fosso del Cavaliere 100, 00133 Rome, Italy; 2 Institute of Atmospheric Sciences and Climate, National Research Council, Corso Fiume 4, 10133 Turin, Italy; 3 Department of Science, Technology and Society, University School for Advanced Studies of Pavia, Piazza della Vittoria 15, 27100 Pavia, Italy; 4 Department of Civil, Environmental and Architectural Engineering, University of Padua, Via F. Marzolo 9, 35131 Padua, Italy

**Keywords:** Hydrogeology, Natural hazards

## Abstract

Land subsidence affects many world metropolises, impacting their infrastructure and population. This work showcases an innovative methodology for exposure-vulnerability rating, hazard quantification and risk assessment that integrates remotely sensed information on ground displacement, land cover and settlement characteristics. Land subsidence-induced deformation and structural stress are quantified within the 15 metropolitan cities of Italy, along with the amount of residential/non-residential infrastructure and population exposed. A total of 1.44 out of 2665 km^2^ urbanised land within the 15 cities is at high risk due to significant angular distortions (and, sometimes, additive threat from horizontal strain) affecting very high exposure-vulnerability infrastructure; for more than 2700 buildings there is high likelihood of already occurred/incipient structural damage. This reference knowledge-base on present-day subsidence-induced risk can inform land and risk management at national scale, and provides a baseline for future assessments to build upon with a look to the next decades and sustainable urban development.

## Introduction

Land subsidence is a widespread threat for many world locations, particularly metropolitan areas built on alluvial plains, where human-induced pressure on urban landscapes and aquifer systems through new urbanization and groundwater exploitation adds onto natural sediment compaction^1–3^. Groundwater provides, indeed, ~ 30% of readily-available freshwater resources globally^4^ and plays a crucial role in addressing global water needs^5,6^. When groundwater withdrawal and natural discharge exceed recharge, aquifer-systems may compact and lose storage, resulting in land subsidence, mostly due to depletion of sandy layers and delayed compaction of clayey aquitards, with rates of a few mm to tens of cm per year^7,8^. This process has been documented in major cities worldwide for decades^9^, with impacts on urban landscapes including development of topographic depressions, tilting and deformation, surface cracking and faulting, apparent uplifting of deeply founded structures, exacerbated risk of flooding in alluvial and coastal areas (especially if combined with sea-level rise)^10^.

By providing robust estimates of ground displacement velocities and time series, satellite Interferometric Synthetic Aperture Radar (InSAR) methods^11^ and, particularly, advanced multi-temporal approaches such as Persistent Scatterers (PS)^12^ and Small Baseline Subset (SBAS)^13^ proved essential to investigate land subsidence^14^, with capabilities ranging from the local to the nationwide^15–19^ and continental^20–22^ scales. To date, InSAR-based studies transforming satellite-derived observations into information layers on exposure, hazard and risk are extremely seldom, yet this is a trending topic in the specialist literature. Differential settlements derived from InSAR have been exploited to quantify angular distortions affecting buildings and to provide insights into surface faulting hazard in major cities of central Mexico^23–27^ and other world countries^28–32^. More recently, research has focused on designing and demonstrating an InSAR-based risk assessment workflow, including approaches for the classification of subsidence-induced hazard and risk levels based on risk matrices^33–40^, as well as the assessment of exposure of communities and urban infrastructure^41,42^, also proving the adaptability of such a methodology to a variety of geographical contexts in the US, Latin America, Europe, Africa and Asia.

Building upon the latest advances in this field, this work: innovates the exposure-vulnerability assessment by designing a novel approach to score this parameter using urban settlement characteristics derived from open global datasets; demonstrates a refined hazard assessment workflow using two structural stress indices well-established in geotechnical engineering, which are derived from open InSAR-based ground displacement datasets; and geospatially combines them through a tailored risk matrix enabling the identification of three increasing levels, from low to high, with the aim to identify highly vulnerable urban infrastructure that might be impacted by differential displacement, and the exposed population. The integration of satellite InSAR data with building characteristics thus represents a significant step forward from displacement velocity-based approaches that are nowadays common in the specialist literature, to actionable risk information that are still rare.

**Fig. 1:**
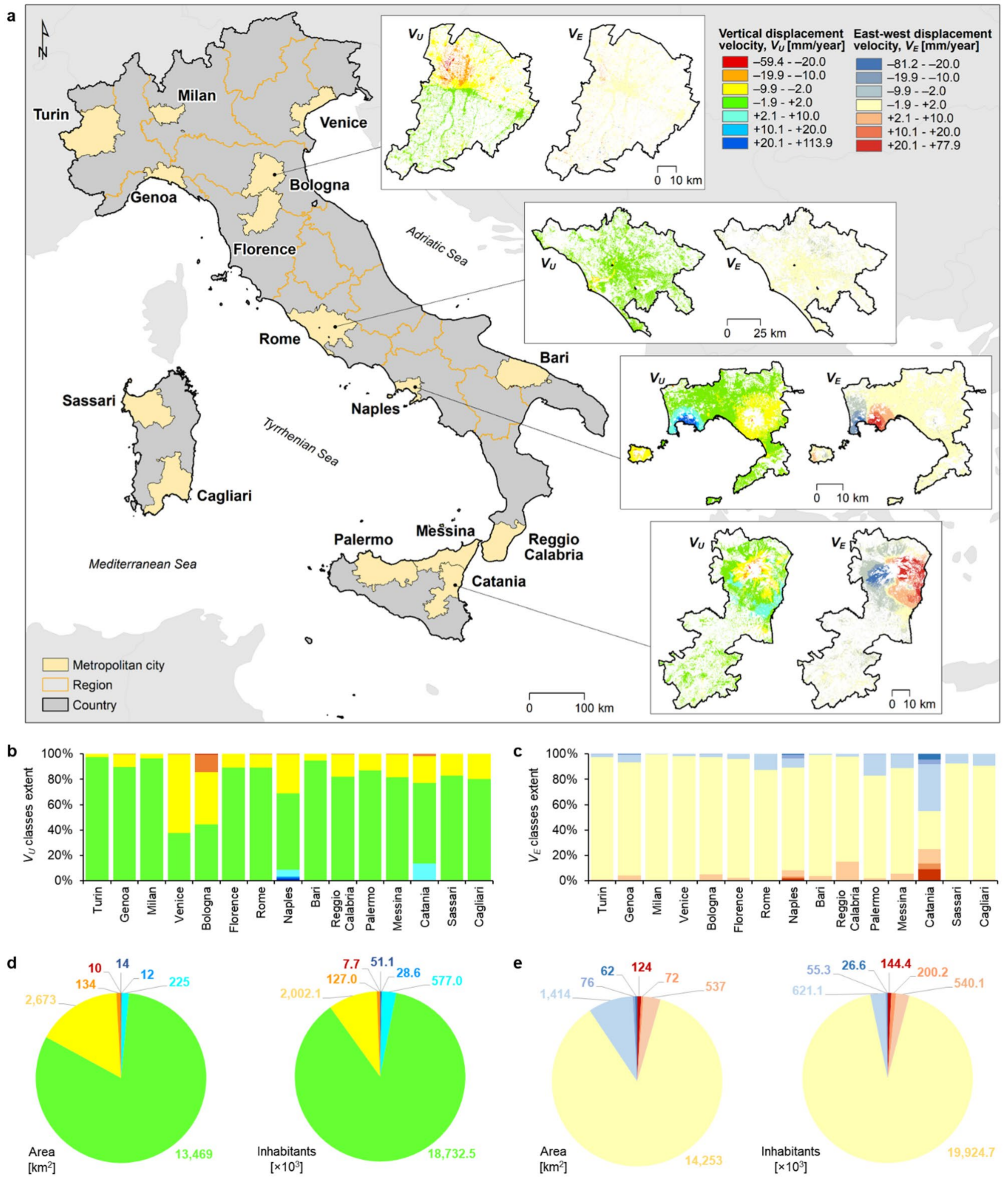
Exposure of Italian metropolitan cities to ground displacement. **(a)** Distribution of the 15 metropolitan cities across the Italian territory, and zoom onto vertical (*V*_*U*_) and east-west (*V*_*E*_) ground displacement velocities for the cities of Bologna, Rome, Naples and Catania; **(b,c)** City-scale and **(d,e)** overall proportion of land and population affected by each vertical and east-west ground displacement velocity class. Administrative boundaries were made available from ISTAT (https://www.istat.it/en), while ground displacement data are based on Copernicus European Ground Motion Service (EGMS) Ortho datasets 2018–2022^75^. Figure **(a)** created using ArcGIS Desktop v.10.6.1 https://desktop.arcgis.com/, and **(b-e)** created using Microsoft Excel 2016 https://www.microsoft.com/it-it/microsoft-365/excel.

The novel character of the work associated with the improved land subsidence risk assessment approach combines with the new insights into exposure, hazard and risk statistics that are derived for the Italian territory, one of the world countries most affected by ground deformation processes. Italy is, indeed, amongst the countries with the greatest subsidence susceptibility and hazard levels^1^, with a number of recognized subsidence hotspots at fluvial and coastal plains (e.g. Po River^43^, Florence-Prato-Pistoia^44^, Tavoliere^45^, Volturno^46^ and Tiber River^47^ plains), mostly involving natural compaction of recent geological deposits accelerated by the effect of intense groundwater withdrawals from aquifers, as well as new urbanization. In 2010, the country was placed among those with the largest estimated groundwater extractions, with a total of 10.4 km³/year pumped from shallow and deep aquifers and destined for agricultural (67%), domestic (23%) and industrial (10%) use^6^. Over the last decade, major cities were supplied with 1.2 km³/year groundwater (i.e. 87% of the total utility supply)^5^. This work therefore focuses on the 15 Italian metropolitan cities (Fig. 1a; Table 1), nerve centre of urban and industrial activities, extending ~ 54,380 km^2^ in total, and hosting > 21.7 million inhabitants (out of the ~ 59.0 censed across the whole country). The exposure of urban land and population to the land subsidence process, ground deformation and its associated hazard, and the resulting risk to urban infrastructure within the 15 cities are assessed with reference to the present-day risk scenario, thus providing a reference knowledge-base with the potential to inform land and risk management at national scale, and a baseline for future assessments to build upon with a look to the next decades and sustainable urban development.

**Table 1:** Metropolitan cities of Italy and their ground deformation-induced risk. Unique IDs, extent and inhabitants censed by ISTAT (https://www.istat.it/en) in 2023 within the 15 metropolitan cities of Italy, with indication of the total number of municipalities belonging to the metropolitan area, and their split based on the maximum hazard and risk levels observed within their administrative land according to the present-day risk assessment: H1 (low), H2 (medium), H3 (high), H4 (very high) hazard; and R1 (low), R2 (medium), R3 (high) risk.

Region	ID	Metropolitan city	Area [km^2^]	Inhabitants (2023)	Number of municipalities							
					Total	H1	H2	H3	H4	R1	R2	R3
Piedmont	201	Turin	6,827	2,198,237	312	217	63	23	9	9	300	3
Liguria	210	Genoa	1,834	813,626	67	19	28	14	6	1	57	9
Lombardy	215	Milan	1,575	3,219,391	133	112	19	2	0	0	132	1
Veneto	227	Venice	2,473	833,703	44	24	13	7	0	0	43	1
Emilia-Romagna	237	Bologna	3,702	1,011,659	55	13	24	16	2	1	50	4
Tuscany	248	Florence	3,514	984,991	41	12	20	8	1	5	35	1
Lazio	258	Rome	5,363	4,216,553	121	72	30	17	2	33	84	4
Campania	263	Naples	1,179	2,969,571	92	53	26	10	3	0	85	7
Apulia	272	Bari	3,863	1,223,102	41	14	17	9	1	0	40	1
Calabria	280	Reggio Calabria	3,210	517,202	97	31	40	24	2	25	71	1
Sicily	282	Palermo	5,009	1,200,957	82	20	38	20	4	9	70	3
	283	Messina	3,266	598,811	108	67	27	9	5	36	67	5
	287	Catania	3,574	1,071,914	58	17	24	12	5	1	51	6
Sardinia	290	Sassari	4,286	315,460	66	42	19	5	0	12	53	1
	292	Cagliari	4,704	543,147	72	28	32	12	0	3	68	1

## Results

### Exposure and vulnerability of Italian metropolitan cities to land subsidence

**Fig. 2:**
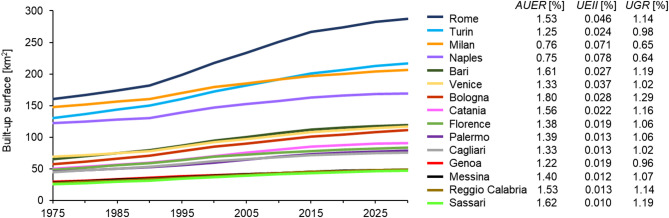
Over fifty years of urban growth across the metropolitan cities of Italy. Temporal evolution of built-up surface extent within each metropolitan city, and associated Annual Urban Expansion Rate (AUER), Urban Expansion Intensity Index (UEII) and Urban Growth Rate (UGR) for the 1975–2025 period. Input data are based on the Global Human Settlement (GHS) Built-up Surface (BUILT-S) dataset^69^. Figure created using Microsoft Excel 2016 https://www.microsoft.com/it-it/microsoft-365/excel.

Since 1975, the 15 metropolitan cities gradually expanded to reach a total of ~ 1700 km^2^ built-up surface as of 2025 (Fig. 2). The temporal trend of urban growth for each city, along with indices such as the Annual Urban Expansion Rate (AUER), Urban Expansion Intensity Index (UEII) and Urban Growth Rate (UGR) (see Methods), highlight a generally slow expansion rate during the last five decades: average yearly percentages of newly urbanized land observed with respect to the 1975 reference (i.e. AUER) are typically around 1.3–1.7%, whereas with respect to the administrative area of each city (i.e. EII) they are mostly below 0.02–0.03%. The UGR metric showcases a similar scenario, with values generally around 1.0–1.1%. Some exceptions are cities such as Bologna and Naples where lower AUER (~ 0.7%) and UGR (~ 0.6%) are associated with higher EII (~ 0.07–0.08%) given their higher proportion of built-up surfaces with respect to the whole administration.

Considering satellite-derived observations of ground displacement for 2018–2022 (see Methods and Fig. 1a), the built-up land exposed to ground instability with annual vertical velocity exceeding ± 2.0 mm/year (either land subsidence or uplift) amounts to a total of 3070 km^2^ across the 15 metropolitan cities (~ 19% of the land covered by satellite observations), against the 13,469 km^2^ remainder that shows rates within the ± 2.0 mm/year range (Fig. 1b, d). Within the displacing areas, the extent of subsiding lands at (negative) rates of more than − 10.0 mm/year is remarkable in Bologna (~ 107.2 km^2^of which 7.2 km^2^ displacing faster than − 20.0 mm/year), Catania (~ 20.8 km^2^), and Rome (~ 3.8 km^2^). Natural and human-induced land subsidence processes are widely recognised as impacting Bologna and Rome, and have been largely studied with InSAR methods^47,48^. In these and other sinking metropolitan cities, if the process persists with significant rates over several years, topographic depressions will continue to gradually develop at subsiding areas, likely resulting in the local exacerbation of fluvial, coastal and/or surface water flooding^33,49,50^. On the other hand, negligible proportions of land within the 15 cities are generally affected by ground uplift with rates of more than + 10.0 mm/year, except in Naples (~ 25.1 km^2^of which 14.3 km^2^ displacing faster than + 20.0 mm/year) and Catania (~ 1.1 km^2^ faster than + 10.0 mm/year) (Fig. 1b, d). In these two cities, the observed ground displacements are mostly driven by volcanic activity (e.g. inflation/deflation at the Phlegraean Fields^51^, and inflation/deflation, compaction of emplaced lava flows and gravity-controlled sliding of the eastern flank at Mount Etna^52,53^).

Looking at east-west displacement rates, the amount of land exposed to annual velocity exceeding ± 2.0 mm/year (either eastward or westward) reaches a total of 2285 km^2^ across the 15 metropolitan cities (~ 14%), against the 14,253 km^2^ showing lower rates (Fig. 1c, e). Catania and Naples are again the cities exposing the largest amounts of land to east-west displacement rates exceeding ± 20 mm/year, with a total of 162.0 km^2^ and 20.5 km^2^ respectively.

While the above figures quantify the amount of land exposed to the different velocity classes, the corresponding amounts of exposed population provide slightly different proportions, as large portions of lands displacing at annual vertical velocity exceeding ± 2.0 mm/year actually involve rural or sparsely populated zones (e.g. in Catania, along the flanks of Mount Etna). A total of about 2.8 million inhabitants (~ 13%) currently reside on land that is displacing beyond ± 2.0 mm/year, against the remainder ~ 19 million inhabitants residing in areas within the ± 2.0 mm/year range (Fig. 1d). Among the former, the amount of population exposed to vertical displacement rates exceeding ± 20 mm/year is ~ 58,800 inhabitants (0.3%). Similarly, the population exposed to east-west rates above ± 2.0 mm/year amounts to about 1.6 million inhabitants (just above 7%), of which ~ 171,000 (0.8%) exposed to rates beyond ± 20 mm/year (Fig. 1e).

**Fig. 3:**
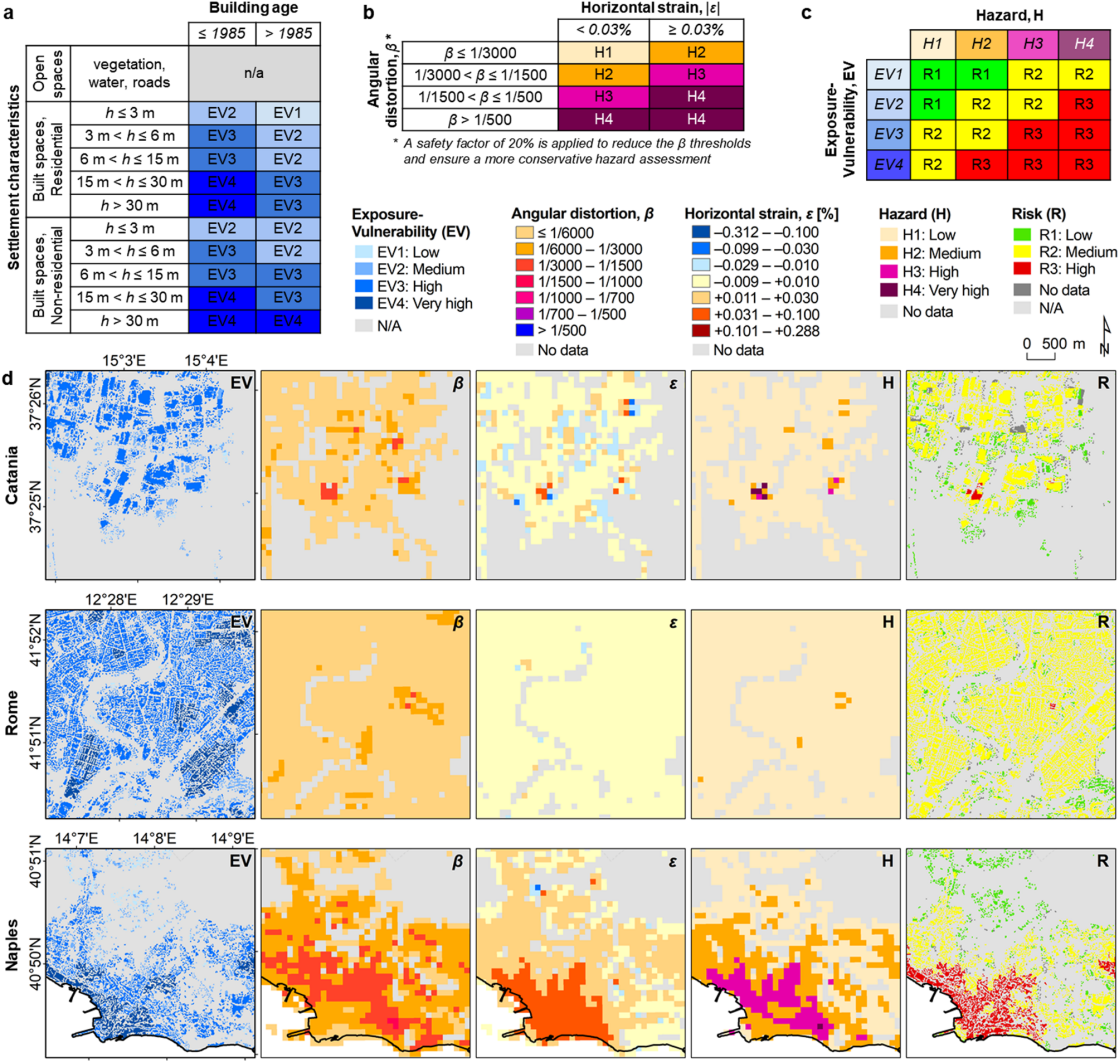
Exposure, hazard and risk to urban infrastructure induced by ground deformation. **(a)** Exposure-Vulnerability (EV) scoring based on settlement characteristics (type, and height, *h*) and age; **(b)** Hazard (H) assessment based on angular distortion (*β*) and horizontal strain (*ε*) induced by ground deformation (differential displacement) on built surfaces; **(c)** Risk (R) matrix allowing the combination of EV and H metrics; and **(d)** zooms onto sample areas in the metropolitan cities of Catania, Rome and Naples. Figures **(a-c)** created using Microsoft Excel 2016 https://www.microsoft.com/it-it/microsoft-365/excel, and **(d)** created using ArcGIS Desktop v.10.6.1 https://desktop.arcgis.com/.

**Fig. 4:**
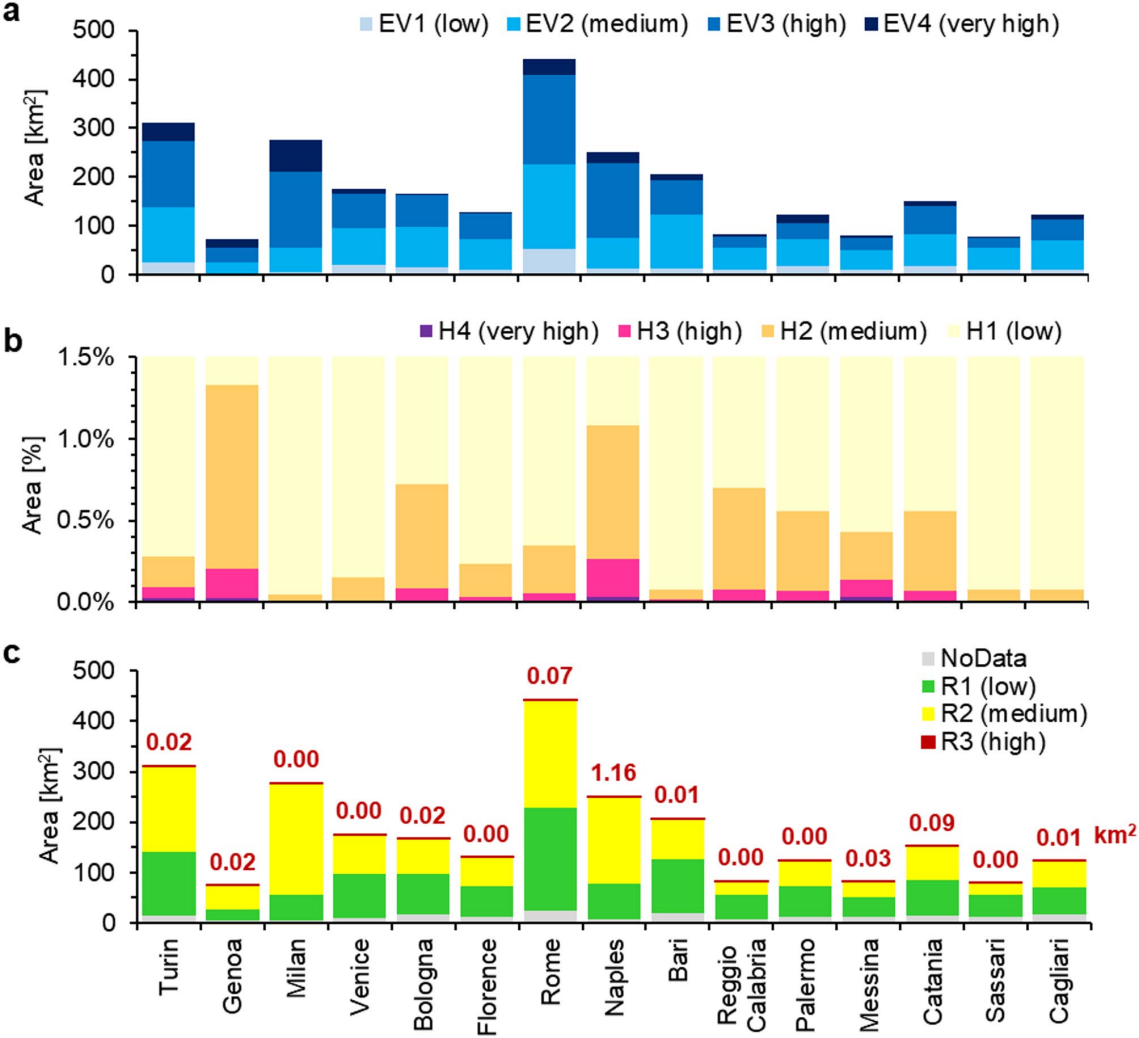
Ground deformation-induced risk in the Italian metropolitan cities. (**a**) Exposure-Vulnerability (EV) of built surfaces, (**b**) hazard (H) induced by ground deformation (differential displacement), and (**c**) resulting risk (R) to urban infrastructure, with indication of the high risk classes. The extent of H classes is expressed as a percentage of the total coverage of ground displacement observations, whereas EV and R classes are represented with their absolute extent. NoData indicates built-up spaces where no hazard information is available. Figure created using Microsoft Excel 2016 https://www.microsoft.com/it-it/microsoft-365/excel.

Since the severity of building damage is controlled not only by the intensity of land subsidence, but also by factors such as the construction year, type, characteristics and maintenance status of the structures and their foundations^54^, an assessment of the exposure-vulnerability of urban infrastructure was carried out, taking into account settlement characteristics (residential/non-residential, and height) and age (pre-/post-1985) of buildings within the metropolitan 15 cities (see Methods and Fig. 3a). Examples of the mapping outputs are provided in Fig. 3d for selected sectors of Rome, Naples and Catania. The proportion of low exposure-vulnerability buildings (EV1) within the 15 cities is generally between 2% (found at Milan, for ~ 6 out of 277 km^2^ built-up spaces) and 14% (Palermo, for ~ 18 out of 123 km^2^). Medium exposure-vulnerability buildings (EV2) typically cover between 17% (Milan, for ~ 48 out of 277 km^2^) and 58% (Sassari, for ~ 45 out of 78 km^2^). Similar proportions are found for high exposure-vulnerability buildings (EV3), extending between 24% (Sassari, for ~ 19 out of 78 km^2^) and 61% (Naples, for ~ 152 out of 251 km^2^). The percentage of buildings associated with the highest exposure-vulnerability metric (EV4) range between 1% (Bologna, for ~ 2 out of 165 km^2^) and 25% (Genoa, for ~ 18 out of 73 km^2^). In terms of absolute extent (Fig. 4a), the cities highlighting the largest extents of EV4 buildings are Milan (~ 65 km^2^), Turin (~ 37 km^2^), Rome (~ 33 km^2^) and Naples (~ 23 km^2^).

### Ground deformation and associated hazard

The leading cause for the development of ground fissures/cracks, surface faulting and structural damage in urban infrastructure is ground deformation that is induced by differential displacement, generating from either ground settlement or heave (e.g. at the boundaries of rapidly subsiding areas, above sharp discontinuities in surface/bedrock geology and aquifer system structure^55,56^), and/or horizontal shrinkage or extension. The amount of angular distortion (*β*)^57^ and horizontal strain (*ε*)^58^ developed over the last 10 years were therefore exploited to assess the probability of occurrence of ground deformation in a particular area and with a specific magnitude, hence hazard^59^ (see Methods and Fig. 3b).

Across the 15 metropolitan cities, *β* ranges between 0 and 0.223% (i.e. 1/448), while *ε* between − 0.312% and + 0.288%. These values quantify the spatial rate of change of differential displacement occurring along the vertical and horizontal (east-west) directions, respectively, across a 100 m horizontal distance; e.g., *β* = 0.20% (i.e. 1/500) refers to 20 cm differential vertical displacement, and *ε* = 0.05% (i.e. 1/2000) to 5 cm differential east-west displacement, across the 100 m distance. Depending on its sign, either positive or negative, *ε* identifies the presence of any tensile or compressive strain that, in structural engineering, respectively indicates “hogging” or “sagging” conditions^56^ (upward or downward bending of a structure under load), and the associated development of cracks at the bottom or top of the affected structure.

The hazard assessment for the 15 cities depicts low hazard levels (H1) across more than 98.5% of the mapped land within each city (Fig. 4b); overall, the greatest proportion of medium (H2), high (H3) and very high (H4) hazard zones is found in Genoa, where hazard is deemed significant across more than 1.3% of the mapped area (~ 6.5 out of 488 km^2^). In terms of absolute extent, Naples (~ 8.5 km^2^) and Palermo (~ 7.0 km^2^) provide the greatest extents of H2 to H4 hazard zones, while the lowest extents are found in Milan (~ 0.4 km^2^) and Sassari (~ 0.9 km^2^). H3 and H4 are generally limited to narrow sectors of the 15 cities, and encompass a total of 9.1 and 1.4 km^2^respectively. The largest extents mapped are 1.8 (H3) and 0.25 (H4) km^2^ in Naples, and 1.5 (H3) and 0.43 (H4) km^2^ in Turin. Some examples of the identified *β* and *ε* and the resulting hazard levels are shown in Fig. 3d.

At municipality level, generally the highest hazard level mapped within the corresponding administrative land is H1 or H2; e.g. 102 out of 121 municipalities (~ 84%) in Rome, 37 out of 55 (~ 67%) in Bologna, 32 out of 41 (~ 78%) in Florence (Table 1). Peaks are observed for Milan (131 out of 133, i.e. ~98%) and Sassari (61 out of 66, i.e. ~92%). On the other hand, the greatest proportions of municipalities reaching H3 and H4 levels are observed in Bologna and Genoa, with a total of 18 out of 55 (~ 33%), and 20 out of 67 (~ 30%), respectively. It is worth noting that in hilly and mountain zones belonging to metropolitan cities such as Turin, Genoa and Reggio Calabria, many localised spots at high hazard are driven by the occurrence of slope instability and mass movements^60^.

### Deformation-induced risk to urban infrastructure

The ‘baseline’ (present-day) assessment of risk to urban infrastructure induced by differential displacement was achieved via geospatial integration of exposure-vulnerability and hazard estimation (see Methods and Fig. 3c). Across the total urbanized area of 2665 km^2^ within the 15 metropolitan cities, a total of 1133 km^2^ low (R1) risk level areas are identified (Fig. 4c). These zones generally cover a large proportion of the mapped areas (~ 30–50%), with peaks at Reggio Calabria (65%) and Sassari (67%); this is considered an acceptable risk level and no specific actions are required in relation to ground deformation-induced risk. Overall, out of the total stock of roughly 5.20 million residential and non-residential buildings within the 15 metropolitan cities according to the EUBUCCO database (see Methods), about 2.76 million (just above 53%) is located in areas at low risk (Table 2).

**Table 2:** Ground deformation-induced risk at buildings within the metropolitan cities of Italy. Number of buildings located in R1 (low), R2 (medium) and R3 (high) risk zones within the 15 metropolitan cities of italy. Building footprints are based on the EUBUCCO database^88^.

Metropolitan city	Number of buildings			
	Total	R1	R2	R3
Turin	733,414	410,068	323,343	3
Genoa	179,346	89,927	89,366	53
Milan	429,940	121,684	308,255	1
Venice	360,661	218,347	142,314	0
Bologna	286,250	175,119	111,111	20
Florence	282,671	148,901	133,770	0
Rome	777,218	481,964	295,226	28
Naples	363,569	125,457	236,294	1,818
Bari	349,862	246,273	103,584	5
Reggio Calabria	224,584	170,342	54,240	2
Palermo	357,500	175,729	181,755	16
Messina	270,675	168,057	102,155	463
Catania	440,943	161,409	279,202	332
Sassari	59,905	37,398	22,507	0
Cagliari	85,419	33,353	52,047	19
Total	5,201,957	2,764,028	2,435,169	2,760

Similarly, medium risk (R2) areas encompass a total of 1351 km^2^ within the 15 cities (Fig. 4c). They typically extend across the vast majority of the administrative land of each city (~ 40–60%), with peaks at Naples (71%) and Milan (81%). Medium risk is considered a relevant level, suggesting that potential structural damage might occur at the urban infrastructure involved; in these areas, tailored monitoring of ground deformation and derived stress indices could be suitable at the building block scale to monitor the process and identify any potential future worsening of the hazard that could impact on the resulting risk level. A total of ~ 2.44 million buildings (~ 47% of the total stock) are located in these areas within the 15 cities (Table 2).

About 1.44 km^2^ of land reveals high (R3) risk levels associated with differential displacement (either land subsidence or uplift), mainly concentrated in narrow sectors exhibiting significant angular distortions (and, in some cases, additive threat from horizontal strain) occurring over very high exposure-vulnerability infrastructure (private/public buildings). These zones generally extend less than 0.01% of the administrative land of each city and < 0.02 km^2^ in total (Fig. 4c); some exceptions are specific sectors within the cities of Naples (~ 1.16 km^2^; the majority of which, in Pozzuoli and Bagnoli), Catania (~ 0.09 km^2^), Rome (~ 0.07 km^2^) and Messina (~ 0.03 km^2^), at locations where the most vulnerable residential and non-residential buildings, with heights often exceeding 15 m, are affected by the greatest differential settlements and induced structural stress (key examples are shown in Fig. 3d). Overall within the 15 cities, a total of 2760 residential and non-residential buildings are located in these high risk areas (Table 2), where there is a high likelihood of already occurred/incipient structural damage. Many of such buildings are located in Naples (1818), Messina (463) and Catania (332), whilst significantly lower amounts are found in other metropolises. Despite the relatively low proportion (~ 0.05%) with respect to the total stock of 5.20 million buildings within the 15 cities, site inspections at single-building scale are recommended in these areas in order to verify the structural health of the buildings and design ad hoc mitigation measures. Thorough validation of the high risk mapping is currently not viable due to absence of a comprehensive structural damage database for the 15 cities, yet the existence of known records of deformation-induced building damage at specific sites substantiates the high risk hotspots observed using this novel risk assessment workflow. For instance, one ~ 5800 m^2^ zone located along Justinian Emperor St. in the centre of Rome is highlighted as at high risk of structural damage, and this corresponds with a cluster of buildings where bending deformation (sagging and hogging), cracks in infill walls, façades and structural elements, as well as other damage, were recorded by independent surveys^61^.

**Fig. 5:**
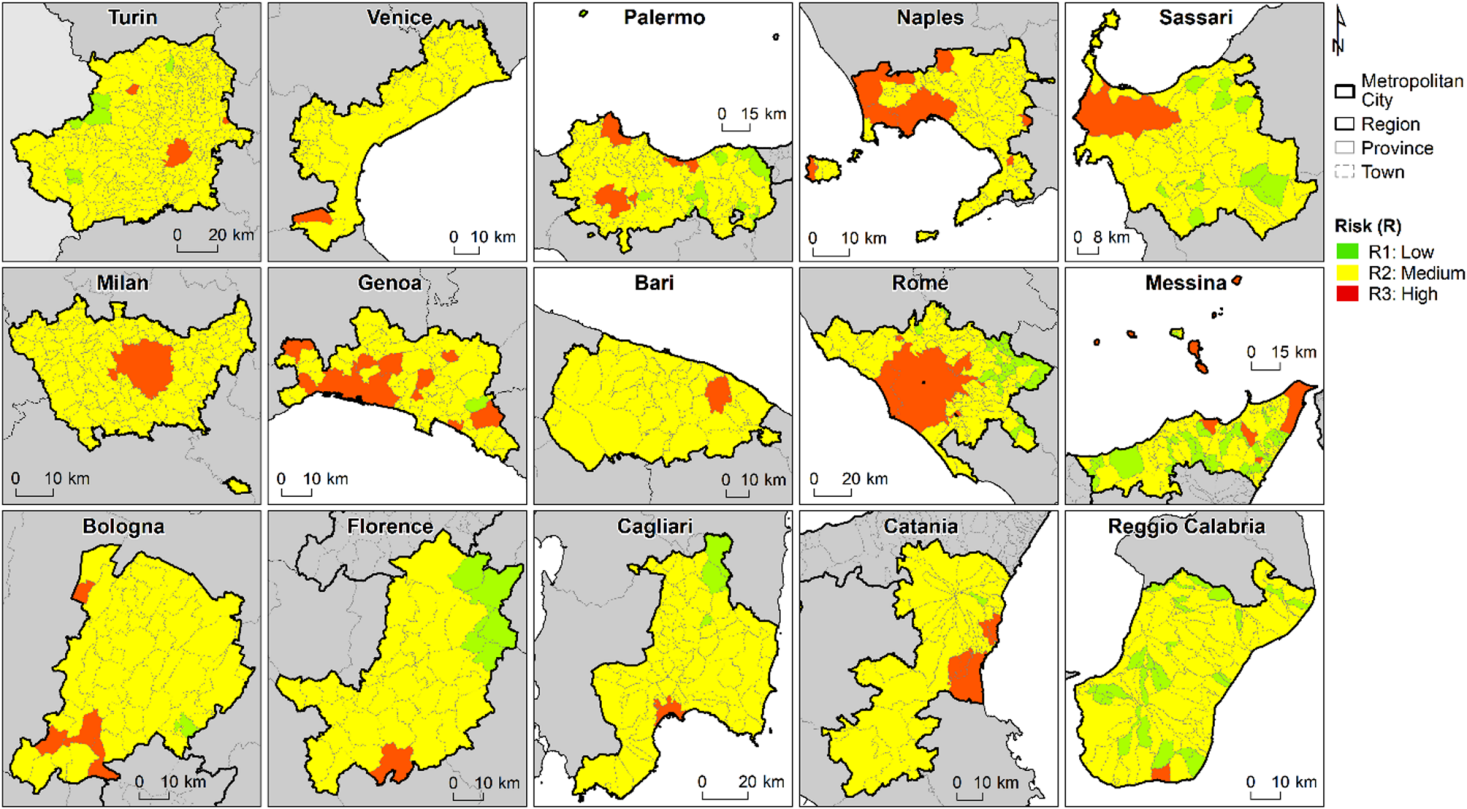
Maximum ground deformation-induced risk at municipality scale within the Italian metropolitan cities. Maximum risk level induced by ground deformation (differential displacement) observed within the municipalities belonging to the 15 metropolitan cities, according to the present-day risk assessment. Table 1 reports the associated statistics. Figure created using ArcGIS Desktop v.10.6.1 https://desktop.arcgis.com/.

Looking at municipality level within each metropolitan city, most municipalities show that the highest risk level mapped within their administrative land is R2, hence moderate risk; e.g. 84 out of 121 municipalities in Rome, 50 out of 55 in Bologna, 35 out of 41 in Florence (Table 1; Fig. 5). Only a few show no more than R1, hence low risk (i.e. no R2 or R3 areas), e.g. nine out of 312 in Turin, nine out of 82 in Palermo, three out of 72 in Cagliari. Nonetheless, the number of municipalities with at least an R3 (high risk) area ranges between one and nine, with the highest values in Genoa (9 municipalities), Naples (7), Catania (6) and Messina (5).

## Discussion

### Technical appraisal of the risk assessment approach

The proposed risk assessment workflow is to be considered alongside warnings associated with its input datasets and technical assumptions (see Methods), namely:

- The exploited urban settlement data do not include information on building foundations, maintenance status or other structural health parameters that may influence their vulnerability. The detailed analysis of such parameters at single building level (if available, for instance from city-scale cadastral databases and recent structural surveys), or the use of more sophisticated approaches (e.g. based on fragility curves^54,62,63^), would enable enhancing the assessment at finer analysis scales.
- During risk assessment, the integration of exposure-vulnerability and hazard layers is tied to the 10 m spatial resolution of the former, yet by acknowledging that latter is embedded at the broader resolution of 100 m; this means that homogeneous hazard levels will be considered across multiple exposure-vulnerability pixels, with no account for any potential variability of subsidence rates and hazard within the 100 m pixels. To account for smaller spatial granularities of hazard information, finer resolution InSAR datasets should be exploited (e.g. calibrated line-of-sight data, combined into vertical and east-west estimates at finer scales^64^).
- InSAR datasets do not provide information on N-S displacement, hence the N-S horizontal strain is not accounted for, whilst it could be significant in structurally-controlled basins delimited by E-W oriented faults. In such cases, other geodetic data (e.g. levelling, or GPS/GNSS), or the use of other image processing methods enabling the estimation of N-S displacements (e.g. pixel-offset tracking^65^, or multiple aperture InSAR^66^), might provide complementary information to enhance the hazard assessment; however, the embedding of these data into the risk assessment workflow is viable only if the geodetic network and/or other processing methods enable the generation of precise N-S displacement maps with spatial resolution comparable with that deriving from the InSAR datasets (e.g. 100 m), thus allowing robust data integration.
- The hazard mapping approach assumes that ground displacement velocities for the 2018–2022 period have affected the observed areas for a total of 10 years. This assumption may result in hazard level underestimation for cities that have been affected by subsidence processes for longer (e.g. Bologna, that experienced human-driven land subsidence since the 1960s^48^); a more extended subsidence history will, indeed, create a higher risk level than the 10 year-referenced calculation suggests. On the other hand, an overestimation of hazard levels may occur for cities that have been affected for shorter periods. To address this, when possible, longer observation periods should be considered (e.g. by exploiting multi-decadal InSAR datasets^64^), as these could allow for a more accurate hazard estimation.
- The risk matrix is designed to output the same level of risk for different combinations of exposure-vulnerability (EV) and hazard (H) pairs; e.g., R2 is identified when EV1 built-up spaces are affected by H3/H4, or also when EV3 areas are affected by H1/H2, and so on. This results in homogeneous risk levels deriving from different input conditions: e.g. large R2 areas in Milan and Florence, where land subsidence affects limited spots and hazard is mostly H1, but exposure-vulnerability of urban structures is EV3-EV4; equally, newer buildings constructed on unstable ground may be ranked the same as old buildings laying on stable ground. By accounting for the EV and H levels generating the risk ranking, and also for additional information from the available input datasets (e.g. settlement characteristics and observed deformation), a more informed judgement on the drivers of the final ranking can be made. Alternatively, more articulated risk matrices outputting greater numbers of risk levels (e.g. R1 to R5, very low to very high risk) could enable to better distinguish different combinations of input EV and H levels.
- Risk maps refer to the risk induced by differential ground displacement, and therefore do not account for land subsidence magnitude and increased development of topographic depressions, or the increased flood exposure, or the loss of coastal land. This aspect also links with the point above, given that similar risk levels are found across cities that appear as mostly stable according to satellite observations but are moderately/highly vulnerable (e.g. Milan), and cities that have been widely impacted by land subsidence over the last decades (e.g. Bologna); in the latter case, despite the large extent of the renown subsiding land, the amount and size of zones affected by differential displacement exceeding the adopted thresholds are relatively limited, hence most of the built-up area is mapped as R2, same as for other cities.
- The accuracy of city-scale and national statistics on the number of buildings located in low, medium and high risk areas is influenced by the completeness, quality and consistency of the input building database; this means that any missing building footprints or spatial inaccuracies in the database will impact the resulting amounts of buildings involved in each risk level. It is therefore pivotal to account for the quality of the exploited building database when attributing a confidence level to the extracted final statistics.

### A look into future risk scenarios

Climate change, population growth, and urban development have been recognized as important drivers of future urban sustainability^67^, and they may further exacerbate land subsidence processes in the next decades. Yet, these factors are often overlooked in the literature and, more generally, in the framework of land management.

The world population is expected to continue increasing and reach ~ 10.4 billion in 2100^68^, with cities and large metropolises further expanding the stock of urbanized land. Over the next 30 years, global water use is projected to grow by 1% per year, and the dependence on groundwater will also rise, exacerbated by climate change (e.g. extreme events, drought and precipitation variability, causing further pressure on aquifers in arid regions) and the induced reduction of surface water availability^5^. Groundwater availability is also expected to decrease, due to reduced natural recharge through precipitation and ground infiltration. Additionally, its quality might be affected by seawater intrusion into coastal aquifers, and pollution via wastewater flooding and seepage from utility networks, posing threats to ecosystems and human health. Globally, the cumulative potential land subsidence area is expected to reach an exposed gross domestic product of ~ 9.2 trillion $ in 2040^1^.

Future research will therefore focus on these dynamics and their strong nexus, in order to assess the knock-on effect of land subsidence and its induced hazard and risk in the next decades, accounting for projected climate change scenarios and future urban development.

## ﻿Methods

### Estimation of urban growth

Urban growth was tracked by exploiting the Global Human Settlement (GHS) built-up surface (GHS-BUILT-S) R2023A dataset^69^, which quantifies the square meters of built-up areas within 100 m ×100 m pixels, every 5 years in 1975–2030. The dataset is derived from Copernicus Sentinel-2 and Landsat multispectral imagery; intermediate epochs that are not covered by direct satellite observations (e.g. data gaps) and future epochs (2025 and 2030) are generated via spatial-temporal inter/extrapolation based on a rank-optimal spatial allocation method^70^.

The amount of built-up surface within each metropolitan city was spatially integrated for each available epoch (*t*_*n*_) to retrieve the corresponding extent of urbanized surface (*A*_*n*_). The extracted series were then exploited to calculate indices that are widely used in urbanization studies^71–74^, namely the Annual Urban Expansion Rate (AUER), Urban Expansion Intensity Index (EII) and Urban Growth Rate (UGR) (Fig. 2):

1 $$\:AUER=\frac{({A}_{n+1}\--{A}_{n})}{{A}_{n}\times{}{t}}$$

2 $$\:UEII=\frac{({A}_{n+1}\--{A}_{n})}{{A}_{MC}\times{}{t}}$$

3 $$\:UGR={\left(\frac{{A}_{n+1}}{{A}_{n}}\right)}^{\frac{1}{t}}-1$$

where *A*_*n*_ and *A*_*n+1*_ indicate the urbanized area at *t*_*n*_ and *t*_*n+1*_ respectively, *Δt* the time span between *t*_*n*_ and *t*_*n+1*_, and *A*_*MC*_ the total extent of the metropolitan city.

### Exposure of urban land and population to land subsidence

To estimate present-day exposure of the 15 metropolitan cities to land subsidence, this work builds upon independent studies on major cities on the US east coast^41^ and China^42^. These extracted areal and demographic statistics by quantifying the amount of land and population within selected classes of InSAR-derived ground displacement velocity. More recently, a study on US metropolises also exploited a more sophisticated way accounting for area-weighted average land subsidence rates, ensuring that estimates better represented the total subsidence-affected area rather than localized hotspots^38^.

Copernicus European Ground Motion Service (EGMS) Ortho 2018–2022 datasets^75,76^ were selected as the key InSAR inputs for the analysis. These provide satellite-derived observations of vertical and east-west ground displacement with millimetre precision^22 ^based on advanced InSAR processing of Sentinel-1 SAR imagery with a temporal sampling following the satellite constellation revisit (6/12 days)^77^. Ortho datasets are 100 m resolution layers derived from the combination of calibrated ascending and descending line-of-sight displacement data (by assuming absence of north-south displacement), referenced to a model derived from global navigation satellite system data^78^. It is worth noting that the spatial coverage of EGMS Ortho datasets mostly follows the distribution of built-up spaces across the investigated areas, as urban structures typically act as good reflectors to the radar signal and, as such, provide the ideal land cover for InSAR methods to perform. Consequently, the dataset provides almost full coverage of the elements at risk with land subsidence estimates and, in turn, hazard information (see also the following sub-sections).

In order to quantify the amount of land and population exposed, the vertical (*V*_*U*_) and east-west (*V*_*E*_) displacement velocities at the EGMS pixels were first reclassified into a symmetric set of 7 classes, bounded by ± 2, ±10 and ± 20 mm/year thresholds (Fig. 1a). The corresponding amount of land affected by each class was then extracted by spatial integration within each metropolitan city (Fig. 1b-c). Similarly, the amount of population residing in each velocity class was computed via spatial intersection with the GHS Population (GHS-POP) R2023A dataset^79^ at 100 m resolution for the 2020 reference year.

### Exposure-vulnerability of built-up areas

Previous land subsidence risk studies accounted for exposure of built-up areas by considering data on the number of properties/houses per square km in Mexico City, Aguascalientes and Morelia (Central Mexico)^33–35^, Lagos (Nigeria)^36^ and US metropolises^38^ or, population density as a proxy for building density in the whole of Iran^39^. For the National Capital Region in India, researchers classified vulnerability based on population, population density and land classification (urban/non-urban)^40^. More recently, a pioneering approach has been demonstrated for three major cities of Italy, to derive a combined exposure-vulnerability metric building upon information on land use/cover and population from the Copernicus Land Monitoring Service (CLMS) – Urban Atlas^64^. The latter accounted for type and attributes of the infrastructure involved (e.g. continuous and discontinuous urban fabric, transport infrastructure, industrial sites), with minimum mapping unit of 0.25 ha in urban areas. This enabled, for the first time, the assessment of vulnerability at the urban block level, although it lacked other key parameters that define building vulnerability, such as height, construction and foundation type, and maintenance status.

In this work, the exposure and vulnerability of urban infrastructure to differential displacement (hence, ground deformation) were assessed based on its spatial distribution, type, height and age. These characteristics provide a proxy for the potential loss that may occur, should infrastructure be affected by differential displacement. Information on the type and height of built surfaces was derived from the GHS Settlement Characteristics (GHS-BUILT-C) R2023A dataset^80^, which delineates the boundaries of human settlements as of 2018, and identifies their morphology and functional use at 10 m resolution, based on the analysis of Copernicus Sentinel-2 imagery. The dataset provides a detailed split between open and built surfaces within human settlements, and information on the type (residential, non-residential) and height (≤ 3, 3–6, 6–15, 15–30, or > 30 m) of built surfaces. These were then geospatially complemented with an estimate of age, as derived from the World Settlement Footprint (WSF^®^) Evolution dataset^81,82^. This outlines the extent of settlement (and non-settlement) areas on a yearly basis in 1985–2015, at 30 m resolution, based on the analysis of Landsat-5/7 data, spectral indices and temporal statistics.

A combined Exposure-Vulnerability (EV) metric was established based on the integration of type, height and age information, with values ranging between low (EV1) and very high (EV4) (Fig. 3a). The metric assumes increasing levels of potential damage that could affect the buildings exposed to the hazard induced by ground deformation, when moving from lower to taller buildings, from residential to non-residential structures (the latter include high-exposure buildings, e.g. hospitals, churches and industrial sheds, which are deemed likely to be more vulnerable to damage), and newer to older constructions (pre-/post-1985; the latter are potentially more vulnerable with respect to new ones that are assumed as complying with recent structural engineering regulations; e.g.^83^). Noticeably, factors such as non-compliance with engineering standards and building codes, the use of low quality construction materials, or lack of maintenance and structural deterioration, all contribute to increasing the vulnerability of urban infrastructure, though require a building-specific knowledge-base to be accounted for; this is outside the remit of the present work, which assumes that the whole stock of new buildings complies with standards and regulations.

To account for the vulnerability of specific types of buildings and construction materials, more sophisticated methods exploiting fragility curves (which depict the conditional probability of reaching/exceeding a given damage state severity level induced by a phenomenon of given intensity) could also be utilised. As these approaches involve the analysis of land subsidence-induced hazard parameters and the induced damage on infrastructure, they are referred to in more detail in the following section.

### Differential displacement and induced hazard

Hazard levels induced by differential displacement (ground deformation) on urban infrastructure were estimated through the computation of the angular distortion (*β*)^57^ and horizontal strain (*ε*)^58^, as derived from the EGMS Ortho InSAR datasets for the 2018–2022 period^75,76^. By assuming that the estimated *V*_*U*_ and *V*_*E*_ affected the metropolitan cities for a period (*Δt*) of 10 years, the total displacement values along the vertical (*d*_*U*_) and east-west (*d*_*E*_) directions, and the resulting total *β* and *ε* were calculated as follows:

4 $$\:{d}_{U}={V}_{U}\times\:\varDelta\:t$$

$$\:{d}_{E}={V}_{E}\times\:\varDelta\:t$$

5 $$\:\beta\:=\frac{{d}_{{U}_{j}-}{d}_{{U}_{i}}}{l}$$

$$\:\epsilon\:=\frac{{d}_{{E}_{j}-}{d}_{{E}_{i}}}{l}$$

where *d*_*Uj*_ and *d*_*Ui*_ (and *d*_*Ej*_ and *d*_*Ei*_) are the vertical (and east-west) displacements of adjacent pixels *j* and *i*, and *l* is their planar distance (in this case, this equals the spatial resolution of the EGMS Ortho datasets, i.e. 100 m).

Depending on the estimated *β* and *ε*, hazard levels were then classified according to a scale ranging from low (H1) to very high (H4) (Fig. 3b), indicating an increasing probability of occurrence of fissuring/fracturing and associated damage of the urban infrastructure.

The threshold values adopted for *β* and *ε* account for geotechnical practice on the allowable settlement of buildings^57,83,84^ and past InSAR-based structural health applications^29–38,41^. In the latter InSAR studies, *β* exceeding 1/3000 (i.e. 0.033%, or also 0.02°) is typically considered as a likely condition for the development of damage (e.g. cracking, tilting, foundation issues) to built-up structures, and is used to distinguish low from medium hazard level; values of 1/1500 (i.e. 0.067%, or also 0.04°) are used to discriminate medium from high hazard levels; values above 1/500 (i.e. 0.200%, or also 0.11°), or 1/150 (i.e. 0.667%, or also 0.38°) are often considered as structurally critical and such to compromise the building serviceability and cause an ultimate limit state, hence are exploited to identify very high hazard. The geotechnical literature highlights that the thresholds are strongly dependent on the type of building/infrastructure, its construction material, characteristics and components (e.g. open/infilled frames, load bearing or brick walls)^56,57,84–86^, yet this information may not be widely available at single building level across whole cities or even countries.

In this work, the thresholds to classify the total *β* over the 10 year-long period into the four hazard levels H1, H2, H3 and H4 were uniformly adopted for any building types/characteristics: 1/3000, 1/1500, and 1/500, along with a safety factor of 20% applied to ensure a more conservative hazard assessment. Following^34^, the hazard scores based on *β* were geospatially complemented with information on presence/absence of significant *ε* that potentially could threaten the structures in addition to *β*. As for *ε*, when its value exceeded ± 0.03% (namely 0.03%, applied to the absolute value of the strain, independently of its direction), a one level increment to the hazard class was accounted for (Fig. 3b).

The reference period of 10 years for the estimation of the cumulated distortions was selected as a trade-off between the 5 year-long satellite InSAR observations and the average likely duration of the estimated ground displacement at the metropolitan cities. While for many major cities such as Bologna, Rome, Florence or Naples there is an established track record of land subsidence observations for a few decades, this is not the case for other cities that might have experienced the process only over the last few years. Similarly, other studies in Spain, Nigeria and the US extrapolated satellite observation periods to refer to lengthier timeframes of 25 years^32,38^, or 10, 35 and 75 years to consider short-, intermediate- and long-term scenarios^36^. In other specific cases when longer observation periods were actually available, such as for the recent city-scale experiments on Bologna, Rome and Florence that were run using 30 years of InSAR data^64^, the exact length of satellite observations enabled a more tailored and accurate assessment.

Regarding the *β* thresholds used to discriminate the hazard levels and the resulting potential damage to infrastructure, other authors demonstrated tailored approaches that build upon the generation of fragility curves, using either subsidence velocity^87^, or the induced differential settlement, *β* and other subsidence-intensity related parameters^62,63^, compared with the observed structural damage at surveyed buildings in Italy and the Netherlands. In particular, two sets of fragility curves for masonry and reinforced concrete structures were established for the historic centre of Como^62^ to account for the different construction materials and consequent vulnerability. Similarly, two sets of curves were identified for single buildings with either shallow or piled foundations in four major Dutch cities^63^. Using Receiver Operating Characteristics (ROC) curves, other authors also run a structural damage prediction test in Granada, building upon *β*-derived potential damage and damage inventory maps^32^. Such approaches could be used to fine-tune the hazard thresholds at the local scale (provided that structural damage records are available, and hence that the fragility and/or ROC curves could be derived), and potentially enable a direct and more robust estimation of risk in terms of expected damage.

### Risk assessment

The deformation-induced risk was finally assessed following the United Nations definition^59^, as the expected loss that could be caused by differential displacement to urban infrastructure. Hazard and exposure-vulnerability information were geospatially integrated via the implementation of a tailored risk matrix, enabling the intersection of the four hazard levels (H1 to H4) and four exposure-vulnerability classes (EV1 to EV4) into 16 possible combinations of likelihood and impact (or also, probability and severity), and the consequent classification of risk in three levels: R1 (low), R2 (medium) and R3 (high), typically represented in green, yellow and red, respectively (Fig. 3c). Such an approach enables the prioritisation of risk resulting from ground deformation, and can potentially provide guidance to land managers and decision-makers in allocating planning and monitoring resources, and implementing any mitigation strategies.

It is worth noting that some limited extents of the exposure-vulnerability zones (typically, this is the case for ~ 5–8% within each metropolitan city of Italy) may not be associated with any risk levels, due to local absence of hazard information. Such zones were classified as NoData (see Fig. 4c).

To quantify the amount of residential and non-residential buildings involved in each risk level, the classified risk maps for each metropolitan city were then superimposed onto the building stock layer from the open EUBUCCO database^88^, which provides > 200 million harmonised individual building footprints across the 27 European Union countries and Switzerland. For Italy, the database includes ~ 20.67 million buildings with a total of 3668 km^2^ footprint area, out of which 5.20 million buildings are located within the 15 metropolitan cities. Statistics on the amount of buildings located in low, medium and high risk areas were extracted by overlapping the risk maps with building footprints from this database, and counting the amount of single polygons intersecting each risk level, within each metropolis. As a proportion of building polygons might be missing from the input layers that were used to generate the EUBUCCO database (namely, regional government cartography and topographic layers, and OpenStreetMap) and/or some polygons were filtered out during the database generation workflow while matching the mapped buildings with global administrative boundaries^88^, the extracted statistics will likely provide a slight underestimation of the actual number of buildings involved in each risk level.

## Data Availability

The administrative boundaries and 2023 census data for the 15 metropolitan cities of Italy are available from the National Institute of Statistics (ISTAT; https://www.istat.it/en). The Global Human Settlement (GHS) POP, BUILT-S and BUILT-C datasets are made available by Copernicus (https://human-settlement.emergency.copernicus.eu), the World Settlement Footprint (WSF^®^) Evolution dataset by the Earth Observation Center (EOC) of the German Aerospace Center (DLR) (https://geoservice.dlr.de), the European Ground Motion Service (EGMS) datasets by Copernicus (https://egms.land.copernicus.eu/), and EUBUCCO dataset by the Mercator Research Institute of Global Commons and Climate Change and the Technical University Berlin (https://eubucco.com). Risk maps and parent layers for the 15 metropolitan cities are openly available for visualization, browsing and querying through SubRISK+ Control Room web platform (https://controlroom.subrisk.eu/).
